# The effects of nature-based interventions on individuals’ environmental behaviors: protocol for a systematic review of controlled trials

**DOI:** 10.3389/fpsyg.2023.1145720

**Published:** 2023-06-02

**Authors:** Dovilė Šorytė, Claudio D. Rosa, Silvia Collado, Vilmantė Pakalniškienė

**Affiliations:** ^1^Faculty of Philosophy, Institute of Psychology, Vilnius University, Vilnius, Lithuania; ^2^Department of Development and Environment, State University of Santa Cruz, Ilhéus, Brazil; ^3^Department of Psychology and Sociology, Universidad de Zaragoza, Teruel, Spain

**Keywords:** environmental behavior, nature-based intervention, nature experience, controlled trial, systematic review

## Abstract

**Background:**

The paper presents the rationale and methods of the planned systematic review to understand the effects of nature-based interventions on individuals’ environmental behaviors. There is ample evidence that experiences in nature not only enhance human well-being but also help promote people’s pro-environmentalism. Nevertheless, synthesized evidence regarding the effects of nature-based interventions on individuals’ environmental behaviors is lacking.

**Methods:**

This protocol follows the Preferred reporting items for systematic review and meta-analysis protocols (PRISMA-P) guidelines. The planned literature search will be conducted by using APA PsycInfo, APA PsyArticles, PubMed, ERIC, Education Source, GreenFILE, OpenDissertations, Scopus, and WEB of Science. In the protocol, we present search strategies for each specific database. Data items that we will seek to obtain from the selected publications are described in detail and cover general information about included studies, information about studies’ methodology and participants, outcomes of the studies, and nature-based and comparative interventions. The outcomes will be behavioral, including aggregated and specific types of environmental behaviors, as well as reported and observed behaviors. Furthermore, the protocol provides a description of the prospective assessment of the risk of bias in both randomized and non-randomized studies. If studies appear sufficiently homogeneous, we will conduct a meta-analysis using the inverse-variance method. Details of the data synthesis are likewise provided in the paper.

**Results:**

Dissemination of the results of the planned review will be carried out via a peer-reviewed open-access journal publication.

**Implications:**

Given the great need to address current environmental issues, understanding what encourages people to act pro-environmentally is critical. It is expected that the findings of the planned review will provide valuable insights for researchers, educators, and policymakers who are involved in understanding and promoting human environmental behaviors.

## 1. Introduction

Nature can contribute to addressing health and social challenges that societies across the globe currently face, including climate change, air pollution, heat stress, noise, low physical activity levels, mental health issues, the health of immigrant populations, and inequality or social exclusion related to urban demography (Hartig et al., 2014; Hordyk et al., 2015; Cohen-Shacham et al., 2016; Ten Brink et al., 2016; Bratman et al., 2019; Rosa et al., 2023a). For instance, human exposure to natural environments is associated with lower levels of stress (Thompson et al., 2012), fewer depressive symptoms (Reklaitiene et al., 2014), better perceived general and mental health (DeVries et al., 2013), children’s cognitive development (Dadvand et al., 2015) and well-being (Brussoni et al., 2017), and lower risk of psychiatric disorders later in life (Engemann et al., 2019). Such findings and many others are summarized in a growing number of systematic reviews and meta-analyses (see, for example, Van den Berg et al., 2015; Tillmann et al., 2018; Corazon et al., 2019; Norwood et al., 2019; Roberts et al., 2019; Weeland et al., 2019; Coventry et al., 2021; Mygind et al., 2021; Rosa et al., 2021, 2023b; Mann et al., 2022; Moll et al., 2022). The synthesized evidence points to the importance of promoting individuals’ experiences with their natural surroundings to enhance their well-being. If populations are aware of these benefits, they might be encouraged to act more pro-environmentally to make sure that natural places are available for people’s reach and function well.

Given the scale of current environmental threats, nature experiences may play a significant role in promoting individuals’ pro-environmentalism, i.e., pro-environmental attitudes and behaviors (Rosa and Collado, 2019). Environmental attitudes are defined as “the collection of beliefs, affect, and behavioral intentions a person holds regarding environmentally related activities or issues” (Schultz et al., 2005, p. 458). Environmental behavior refers to actions that influence the sustainability of nature (Schultz and Kaiser, 2012). Pro-environmentalism is often linked to the nature experiences in childhood (e.g., D’Amore and Chawla, 2020). Retrospectively measured childhood contact with the natural world shows positive relationships with adults’ environmental attitudes (Wells and Lekies, 2006; Broom, 2017) and behaviors (Wells and Lekies, 2006; Broom, 2017; Rosa et al., 2018; Molinario et al., 2020). In a longitudinal study conducted by Evans et al. (2018), it was found that there is a positive association between childhood time spent outdoors and increased environmental behavior later in young adulthood. Moreover, individuals’ experiences with nature and their environmental behaviors can be directly and indirectly related (Collado et al., 2015). The relationship might be mediated by several factors, including connectedness to nature (Otto and Pensini, 2017; Martin et al., 2020), environmental attitudes (Collado et al., 2015; Collado and Corraliza, 2015), and biocentric values (Larson et al., 2011). Though associations between experiences in nature and environmental behavior are complex, nature-based interventions could not only improve individuals’ well-being but also act as an effective strategy to promote their environmental behaviors.

To date, there are several published reviews examining links between nature exposure and individuals’ pro-environmentalism. These analyses include an overview of various research approaches used to investigate the relationship between experiences in nature and human pro-environmentalism (Rosa and Collado, 2019) as well as—more specifically—an examination of the relations linking nature-based tourism and tourists’ environmental knowledge, attitudes, and behaviors (Ardoin et al., 2015). In addition, as already noted, considerable attention has been given to the links between nature experiences in childhood and the further development of pro-environmentalism. These analyses include an examination of the associations between nature exposure in childhood and the subsequent development of environmental attitudes and behaviors (DeVille et al., 2021), developmental research related to climate change (Hahn, 2021), and a review of interventions aimed at increasing nature connection in children (Barrable and Booth, 2020). The evidence derived from these reviews until now points to a positive relationship between nature experiences and pro-environmentalism. Nonetheless, to our knowledge, there are no systematic reviews assessing primary studies that evaluated the effects of nature-based interventions on individuals’ environmental behaviors. Environmental behavior is our main focus because behavioral change is the ultimate goal of all the efforts directed at strengthening pro-environmentalism (Rosa and Collado, 2023). Knowledge gaps include identifying specific elements of nature contact or specific types of nature-based activities that could be most effective to encourage environmental behaviors (Rosa and Collado, 2019; DeVille et al., 2021). As an example, activities like walking, playing, or hiking in the wilderness during childhood may be positively associated with the development of pro-environmentalism (Wells and Lekies, 2006). However, such studies are mostly retrospective and do not allow for drawing causal conclusions. Furthermore, efforts have been made to evaluate what environmental behavior outcomes result from individuals’ psychological connection with nature (i.e., the extent to which people see themselves as part of nature or the subjective sense of “oneness” with nature) (Mackay and Schmitt, 2019; Barragan-Jason et al., 2022, 2023) and physical connection with nature (i.e., contact with the natural world) (Barragan-Jason et al., 2023). However, little is known about the specific effects that nature-based interventions have on individuals’ environmental behaviors, as compared to other types of interventions.

This paper aims to fill the gap of the synthesized knowledge about the effects of nature-based interventions on individuals’ environmental behaviors. With the planned review, we aim to answer the following research question: What are the effects of nature-based interventions on individuals’ environmental behaviors compared to any other type of intervention or no intervention?

## 2. Methods and analysis

This protocol is prepared following the PRISMA-P 2015 initiative, i.e., the preferred reporting items for systematic review and meta-analysis protocols (Moher et al., 2015; Shamseer et al., 2015).

### 2.1. Eligibility criteria

Study eligibility criteria by study characteristics and report characteristics are presented in Table 1.

**Table 1:** Study characteristics and report characteristics to be used as study eligibility criteria for the review.

	Eligibility criteria	Description
	*Study characteristics*	
1.	Participants	Individuals of various ages. Both normative and clinical samples would be included.
2.	Interventions/settings	Interventions of varying type and length that are based in natural settings, i.e., any place that includes natural elements, such as animals and plants. Following Kuo et al. (2019), nature exposure includes experiences of nature not only in the wilderness but also within human-built contexts. Thus, city parks, school yards or similar places will also be considered natural settings. However, natural elements found indoors (e.g., pictures of nature in the classroom or trees visible through the window) will be assigned to indoor settings, and studies of this type will be excluded from the analysis.
3.	Comparators	Any other type of intervention (e.g., indoor-based interventions) or control groups without an intervention.
4.	Outcomes	Outcomes of various environmental behaviors, including both aggregated (e.g., environmental, ecological, or conservation behavior), and specific actions (e.g., recycling, water conservation, or re-use behavior), as well as both reported (i.e., self-reports by study participants or other-reports/peer ratings) and observed behaviors (i.e., using field observations by trained observers or laboratory observations). Outcomes related to individuals’ health, well-being, or development will be excluded.
5.	Study design	Quantitative research design; randomized and non-randomized controlled trials.
6.	Time frame	There will be no restrictions on the length of follow-up.
	*Report characteristics*	
7.	Years considered	Study publication years range from the earliest possible.
8.	Language	Studies published in any language. In case translation is required, translation tools will be used. However, if the review team is not able to translate the study reliably, such a study will be excluded with a clear recording.
9.	Publication status	Studies published in peer-reviewed sources, excluding literature reviews (nevertheless, reviews identified through the searches will be saved for relevant reference lists). Grey literature (e.g., dissertations and conference papers) will also be included. Moreover, studies will not be excluded based on publication status.

### 2.2. Information sources and search strategy

The literature search will be conducted in the following electronic databases: APA PsycInfo, APA PsyArticles, PubMed, ERIC, Education Source, GreenFILE, OpenDissertations, Scopus, and WEB of Science. To supplement the search, we will also scan the reference lists of included studies and literature reviews with similar research questions. To not miss out on more recent studies, we plan to update the search once again just before the submission of the systematic review. In addition, the reviewers will scan their files in an effort to ensure that no relevant study has been missed. Such studies will be recorded.

To create a search strategy that would be balanced between sensitivity and precision, we followed a method proposed by Bramer et al. (2018). Our “key concepts” or search elements are “nature-based” and “environmental behavior.” Another element used in the search strategy is specific research design, i.e., randomized and non-randomized controlled trials. APA PsycInfo (in EBSCOhost) was chosen as an appropriate database to start with because it is a comprehensive database of interdisciplinary psychological research and it uses a thesaurus. We first identified appropriate terms in the APA Thesaurus of Psychological Index Terms, starting from the most relevant terms related to our search elements, followed by “exploding” selected thesaurus terms. Because APA PsycInfo does not generate lists of synonyms in the thesaurus, we could not use these as free-text terms. For this reason, relevant reviews were used to find narrower terms (as free-text terms) in relation to natural environments (Tillmann et al., 2018; Corazon et al., 2019; Norwood et al., 2019; Roberts et al., 2019; Weeland et al., 2019; Mann et al., 2021; Moll et al., 2022) and environmental behavior (Bamberg and Möser, 2007; Mackay and Schmitt, 2019). Terms related to specific research design were based on the search strategy developed by Roberts et al. (2019). We further followed the guidance on optimizing the search, i.e., identifying missed free-text terms and missed thesaurus terms (Bramer et al., 2018). Lastly, the search strategy was translated to other databases. For each specific database that has a thesaurus, thesaurus terms were adapted. The planned search strategy for each specific database is presented in Supplementary File 1. Where possible, the searches will be restricted to the population group of humans.

### 2.3. Study records and selection process

Literature search results will be uploaded to Covidence—a systematic review data management platform. Two researchers will independently screen titles and abstracts of publications selected according to the study inclusion criteria. After screening titles and abstracts, two researchers will independently screen the full-text articles. Duplicate publications and data from multiple reports of the same study (if any) will be identified by juxtaposing author names, specific details, and settings of interventions. In case of multiple reports, we will choose one report as a primary and will not include other reports as separate units. However, we will not discard other (secondary) reports if they contain additional outcome measures or other valuable information regarding the study (Lefebvre et al., 2022).

Any disagreements about the inclusion of particular publications will be resolved through discussions. If disagreements between two reviewers cannot be solved via a discussion, a third author will be involved.

### 2.4. Data collection process and data items

Data from selected publications will be extracted by one reviewer with verification by another to reduce bias and errors in data extraction. *A priori* form (see Table 2) is prepared for this purpose. If needed, we will contact the studies’ authors by email (a maximum of two email attempts) to solve any uncertainties or to ask for any necessary missing information. Discrepancies will be resolved through discussions. If the attempts to contact the studies’ authors are unsuccessful, unclear or incomplete information will be specified in the completed review. In case data synthesis is possible, imputation methods will be applied to handle missing data (see Section 2.7).

**Table 2:** *A priori* data extraction form.

	Data items	Description
1.	Authors	Names of study authors.
2.	Study title	Title of the study.
3.	Country	Country where the intervention was implemented.
4.	Age	Participants’ age by years: mean age and range.
5.	Sample size	The number of participants who participated in the intervention (and in a comparison group).
6.	Gender	Percentage of females who participated in the intervention.
7.	Was the sample normative	Samples should be indicated as normative or clinical; if clinical, the group should be specified.
8.	Intervention setting	Particular natural settings where the intervention took place, and settings of the comparative intervention.
9.	Intervention activities	Information on specific activities that were carried out during intervention and its providers (i.e., who carried out the activities), as well as possible co-interventions that were provided to the participants and were not nature-based (e.g., environmental education classes).
10.	Comparative intervention	Information on analogous aspects of comparative intervention, i.e., specific activities and the providers.
11.	Intervention timing	Length, duration, and frequency of intervention.
12.	Outcomes	Type of environmental behaviors and other outcomes, if any.
13.	Study design	Controlled studies that involve randomization will be assigned to randomized trials, and controlled studies that do not involve randomization will be assigned to non-randomized trials.
14.	Measurement instruments	Information on environmental behavior measures that were used to assess the effect of the intervention of interest.
15.	Follow-up timing	Length and number of follow-up measurements.
16.	Change in environmental behavior	Change in environmental behavior from baseline to post-intervention time point, or post-intervention environmental behavior estimates if behavioral change is not reported. We will collect both estimates of environmental behavior and SDs of those estimates before and after the intervention.
17.	Dropouts	The number of participants who withdrew (with the indication of reasons).
18.	Adverse events	Information about the possible adverse events that occurred during the nature-based intervention (e.g., participants got hurt).
19.	Miscellaneous	Other important comments from study authors.

As presented in Table 2, we will obtain data items that cover general information about included studies (titles, authors, and countries), information about study participants (age, sample size, gender, and whether the sample was normative), interventions (settings, timing, providers, and specific activities carried out), the comparative interventions (the analogous information about comparison groups), and outcomes (types of environmental behaviors and change in the behaviors, as well as other outcomes, if measured). Moreover, we will obtain data regarding the studies’ methodology (study design, instruments, and follow-up timing).

### 2.5. Outcomes

The outcomes will be behavioral, i.e., various environmental behaviors that include aggregated and specific environmental actions, reported and observed behaviors (see Table 1). As indicated above, environmental behavior refers to actions that influence the sustainability of nature (Schultz and Kaiser, 2012). Actions that exert a positive influence are considered pro-environmental behaviors, while actions that influence the sustainability of nature negatively are harmful environmental behaviors.

Acceptable outcome measures for the prospective review involve quantitative research tools. In general, most research concerning environmental behavior relies on self-reports (Steg and Vlek, 2009). There is a great variety of self-report measurements, including interviews, questionnaires, single-item instruments and multi-item scales, diary procedures, and ecological footprint measures, and they target various behavioral properties, such as frequency of engaging in environmental behavior or whether individuals engage in a particular behavior or not (Lange and Dewitte, 2019). One of the most well-known and widely used self-report instruments is General Ecological Behavior (GEB) scale (Kaiser and Wilson, 2004). It is considered the best-established measure for assessing domain-general behavior (Lange and Dewitte, 2019), i.e., a composite measure of various conservation behaviors. Though widely used, self-reports have limitations regarding the validity of such instruments (Steg and Vlek, 2009; Kormos and Gifford, 2014; Lange and Dewitte, 2019). More objective measures applied in the field can be broadly classified into device measurements (usually by using meter readings, e.g., readings of electricity consumption), peer ratings (i.e., ratings by people who are close to participants), and observations made by trained observers (Kormos and Gifford, 2014). Moreover, in contrast to field observations by trained observers, a higher degree of experimental control can be achieved *via* laboratory observations (Lange and Dewitte, 2019), like the recently developed Pro-Environmental Behavior Task (PEBT) (Lange et al., 2018). To sum up, given the great diversity of environmental behavior measurement tools, we expect to find very different research measures for the behavioral outcomes under consideration, and limit our systematic review to the quantitative ones. Importantly, when evaluating interventions that promote environmental behavior, field observations or observations of behavioral products (i.e., instead of observing the performance of behavior, the behavioral residues are targeted) should be considered the most appropriate methodology (Lange and Dewitte, 2019).

When a study includes several pre- and post-intervention tests, we will consider the pre-test closer to the start of the intervention and the post-test closer to the end of the intervention. We do this to improve comparability between studies, and because such information can be found in most of the studies. In addition, we will also select the longest (latest) indicated follow-up in each selected study. Such information will be clearly described in the review and will allow for capturing the final behavioral changes, if found. However, we are aware that this may as well induce a lack of consistency across studies (Higgins et al., 2022). A maximum of two post-intervention time points could therefore be retrieved.

### 2.6. Risk of bias in individual studies

To assess the risk of bias in randomized studies, we will use RoB 2, i.e., an updated version of the Cochrane risk-of-bias tool (Sterne et al., 2019). Bias domains that will be included in the evaluation process are as follows: bias arising from the randomization process; bias due to deviations from intended interventions; bias due to missing outcome data; bias in the measurement of the outcome; bias in the selection of the reported result, and an overall bias. Within each domain, the reviewers will answer signaling questions that will lead to the judgments of “low risk of bias,” “some concerns,” or “high risk of bias” (Sterne et al., 2019). To evaluate the risk of bias in non-randomized studies, another tool (ROBINS-I) will be applied. According to Sterne et al. (2022), many features of ROBINS-I are shared with the RoB 2 tool because both focus on specific results, have a fixed set of domains of bias, include signaling questions, and lead to an overall risk-of-bias judgment. In the case of ROBINS-I, the judgments can be “low,” “moderate,” “serious,” or “critical” risk of bias, and the key concerns are confounding, selection bias, information bias, and reporting bias (Sterne et al., 2022). The risk of bias assessment results will be summarized in tables and text in the completed review.

Evaluation of the risk of bias will be undertaken by two reviewers independently. If any disagreement arises, the third reviewer will be the arbitrator. The reviewers will not be blinded to the studies. Among the authors, some reviewers have previous risk of bias assessment experience.

### 2.7. Data synthesis

If studies are sufficiently homogeneous in terms of participants, intervention, comparator, outcome, and follow-up times, we will conduct a meta-analysis using the inverse-variance method. Studies that are similar enough will be grouped. Assuming studies would differ regarding participants’ characteristics as well as in the implementation of interventions, the random-effects method will be applied (Borenstein et al., 2010). However, in case very few studies (i.e., 2–4) are found, the fixed-effects method would be used instead because it is considered more appropriate for situations when heterogeneity cannot be reliably evaluated (this is the case if only a few studies are available) (Bender et al., 2018). Regarding effect measures, Hedges’ *g* for continuous outcome data and risk ratios for dichotomous outcomes (with 95% confidence intervals) will be used. As mentioned, in case of missing data, the authors of the studies will be contacted. However, if such attempts are unsuccessful, imputation methods will be applied. Specifically, we would input standard deviation (SD) from similar included studies, using their average or median SD values (Higgins et al., 2022). Sensitivity analysis would be applied to explore the impact of the imputation method. Furthermore, for missing mean values, a formula proposed by Wan et al. (2014) would be applied. It is based on the lower quartile, median and upper quartile summary statistics.

If multiple measurement instruments are used to register environmental behavior within the same study, we plan to apply a decision rule approach for selecting one outcome (effect estimate) (McKenzie et al., 2022). Where possible, we will specifically consider the methodological aspect (psychometric characteristics of the measures) and select the most appropriate one. For instance, observed measures would be prioritized over both self-reported and other-reported (ratings by peers) measurement tools because field observations are considered a more appropriate methodology in the case of intervention evaluation (Lange and Dewitte, 2019). When there is more than one group of participants in a study, and environmental behavior is registered by different measures in each group, these different outcomes will be considered. As already specified, in the case of different pre- and post-intervention time points within the same study, we will consider the pre-test closer to the start and the post-test closer to the end of the intervention, together with the longest follow-ups.

We anticipate that there might be significant variability in the studies’ design and outcome measurement instruments. Thus, we will tabulate studies’ characteristics and assess them visually by comparing PICOS across studies to evaluate heterogeneity. Moreover, to evaluate statistical heterogeneity, I^2^ estimate will be used. According to Deeks et al. (2022), *I*^2^ ranging from 0% to 40% indicates that heterogeneity might not be important, and 30% to 60%—that heterogeneity is moderate, while 50% to 90% and 75% to 100%, respectively represents substantial and considerable heterogeneity. Moreover, if possible, subgroup analysis by participants’ age (i.e., children, adolescents, and adults) will be implemented to investigate heterogeneity.

In case quantitative synthesis is not possible, qualitative (narrative) synthesis will be presented in tables and text that summarize and explain the findings of included studies. In the tables, we will provide characteristics of included studies, despite their risk of bias. Given the developmental differences between children, adolescents and adults, the description of the outcomes for the three groups will be provided separately: children (up to 12 years), adolescents (13–17 years), and adults (18 years and older). We will first describe the results of the randomized controlled trials, followed by describing the findings of non-randomized studies. We plan to report studies of any level of risk of bias with a clear indication of whether the risk was low, moderate, or high/critical. Finally, to assess and summarize the resulting evidence’s certainty, the Grading of Recommendations Assessment, Development and Evaluation (GRADE) approach will be applied considering the domains of risk of bias, inconsistency, indirectness, imprecision, and publication bias (Schünemann et al., 2022). Following this approach, the assessment will be based on the four levels of certainty, i.e., high, moderate, low, and very low.

Publication bias assessment would be based on the comparison of published and similar unpublished studies by conducting a subgroup analysis (Boutron et al., 2022).

## 3. Discussion

Given the pressing need to address current environmental issues, such as climate change (IPCC, 2022), understanding the determinants of people’s environmental behavior is crucial. Previous findings suggest that experiences with nature are linked to environmental behaviors (Wells and Lekies, 2006; Larson et al., 2011; Collado and Corraliza, 2015; Broom, 2017; Otto and Pensini, 2017; Rosa et al., 2018; Molinario et al., 2020). However, to our knowledge, the extent of the effect of nature-based interventions on people’s environmental behavior has not yet been systematically examined (see, however, analyses on related topics, e.g., Mackay and Schmitt, 2019; Barragan-Jason et al., 2022, 2023). Moreover, the associations that link experiences in nature and environmental behaviors are not yet fully understood (Ardoin et al., 2015; Rosa and Collado, 2019; DeVille et al., 2021). This protocol presents the rationale and methods of the planned systematic review aimed at understanding the effects of nature-based interventions on individuals’ (including children, adolescents, and adults) environmental behaviors. Such knowledge could be relevant for researchers, educators, and policymakers, who are involved in understanding and promoting environmental behaviors and sustainable development in general.

Results from the prospective review could be valuable in a number of ways. First, at the political level, special attention has been given to the so-called nature-based solutions which refer to the cost-effective solutions that use ecosystems to effectively address societal challenges and simultaneously provide environmental, social, and economic benefits (Cohen-Shacham et al., 2016; European Commission, 2021). Despite its significance, the potential of nature-based solutions for a transformative change remains unexplored. This is especially relevant considering that one of the spheres of this transformation relates to behavior (i.e., changes in individuals’ habits and lifestyles that are positive for the environment; Palomo et al., 2021). The planned systematic review could thus contribute to the insights into what kind of nature-based programs are particularly important in driving this behavioral change. Second, the synthesized evidence about the role that nature-based interventions play in promoting environmental behaviors could be a basis for further development of interventions that are based in natural settings. More in-depth research is still needed to clarify the conditions under which specific outdoor learning forms are most beneficial for various target outcomes (Mann et al., 2022). Such knowledge could be applied by educators who use (or consider using) natural environments in their practice with the aim to promote individuals’ environmental behaviors. Lastly, the synthesized knowledge could be of interest to researchers who seek to advance scientific knowledge on the effect of nature-based interventions on environmental behaviors, or to those who seek to implement and evaluate the impact of nature-based programs on environmental behavior. Ultimately, individuals’ contact with nature could potentially contribute to fulfilling several crucial goals at once: help improve people’s health and well-being, and enhance pro-environmentalism.

## 4. Ethics and dissemination

Considering that the planned systematic review will include secondary data analysis only, no ethical approval will be sought. Dissemination of the results of this review will be carried out via a peer-reviewed open-access journal publication.

## Data availability statement

The original contributions presented in the study are included in the article/Supplementary material, further inquiries can be directed to the corresponding author.

## Author contributions

DŠ drafted the work. CR, SC, and VP provided substantial contributions to the conception and design of the work, and revised it critically. All authors contributed to the article and approved the submitted version.

## Funding

CR is receiving a scholarship from Coordenação de Aperfeiçoamento de Pessoal de Nível Superior (finance code: 001). No financial or other support was provided for this review.

## Acknowledgments

The authors would like to thank the reviewers for their valuable help in improving the manuscript.

## Conflict of interest

The authors declare that the research was conducted in the absence of any commercial or financial relationships that could be construed as a potential conflict of interest.

## Publisher’s note

All claims expressed in this article are solely those of the authors and do not necessarily represent those of their affiliated organizations, or those of the publisher, the editors and the reviewers. Any product that may be evaluated in this article, or claim that may be made by its manufacturer, is not guaranteed or endorsed by the publisher.
