# Supplementary material for: The effects of nature-based interventions on individuals’ environmental behaviors: protocol for a systematic review of controlled trials
Source: Front Psychol. 2023 Jun 2;14:1145720. doi: 10.3389/fpsyg.2023.1145720 (PMC10275608; doi:10.3389/fpsyg.2023.1145720)
Supplement: Supplementary file 1 [file Data_Sheet_1.docx]

Supplementary File 1

The effects of nature-based interventions on individuals’ environmental behaviors: protocol for a systematic review of controlled trials

Dovilė Šorytė*, Claudio D. Rosa, Silvia Collado, Vilmantė Pakalniškienė

*** Correspondence:** Dovilė Šorytė: dovile.soryte@fsf.vu.lt

# Supplementary Tables

The planned search for the APA PsycInfo database (via EBSCOhost).

| **Search string** |
| --- |
| (((DE "Nature (Environment)") OR AB (“natur* environment*” OR “natur* setting*” OR “natural outdoor environment*” OR “exposure to natur*” OR “nature exposure” OR “contact with natur*” OR “nature interaction*” OR “nature experience*” OR “nature assisted” OR “nature based” OR “nature therapy” OR ecotherapy OR “therapeutic landscape*” OR “time in natur*” OR “time spent in natur*” OR park OR parks OR forest OR forests OR wood OR woods OR garden OR gardens OR horticultur* OR outdoor* OR outside OR “green space*” OR greenspace* OR greenness OR “green setting*” OR “blue space*” OR seaside OR “sea side” OR wilderness OR vegetation OR tree* OR bush* OR woodland* OR allotment* OR countryside OR “nature school*” OR “experience* with natur*” OR “natural world” OR “nature contact” OR “engagement with natur*”)) AND ((DE "Pro Environmental Behavior" OR DE "Conservation (Ecological Behavior)") OR AB (“environmental behavio*” OR “environmental action*” OR “environmentally responsible behavio*” OR “ecologically responsible behavio*” OR “environment* friendly behavio*” OR “sustainable behavio*” OR “sustainable action*” OR “pro environmental collective action*” OR “environmental activism” OR “environmental stewardship” OR “conservation behavio*” OR “environmental consumer behavio*” OR “ecological consumer behavio*” OR “sustainable consumption” OR “green consumer behavio*” OR “ecological behavio*” OR “environment* friendly consumer behavio*” OR conservationism OR “environment protect* behavio*” OR “green consumption” OR “nature protective behavio*”)) AND ((DE "Intervention" OR DE "Experimental Design" OR DE "Experimental Methods" OR DE "Quasi Experimental Methods" OR DE "Randomized Controlled Trials") OR AB (intervention OR experiment OR randomized OR crossover OR “pre post” OR comparison OR controlled OR control))) |

*Note:* Search limited to the Population group of humans.

The planned search for the Scopus database.

| **Search string** |
| --- |
| (TITLE-ABS(“natur* environment*” OR “natur* setting*” OR “natural outdoor environment*” OR “exposure to natur*” OR “nature exposure” OR “contact with natur*” OR “nature interaction*” OR “nature experience*” OR “nature assisted” OR “nature based” OR “nature therapy” OR ecotherapy OR “therapeutic landscape*” OR “time in natur*” OR “time spent in natur*” OR park OR parks OR forest OR forests OR wood OR woods OR garden OR gardens OR horticultur* OR outdoor* OR outside OR “green space*” OR greenspace* OR greenness OR “green setting*” OR “blue space*” OR seaside OR “sea side” OR wilderness OR vegetation OR tree* OR bush* OR woodland* OR allotment* OR countryside OR “nature school*” OR “experience* with natur*” OR “natural world” OR “nature contact” OR “engagement with natur*”) AND TITLE-ABS(“environmental behavio*” OR “environmental action*” OR “environmentally responsible behavio*” OR “ecologically responsible behavio*” OR “environment* friendly behavio*” OR “sustainable behavio*” OR “sustainable action*” OR “pro environmental collective action*” OR “environmental activism” OR “environmental stewardship” OR “conservation behavio*” OR “environmental consumer behavio*” OR “ecological consumer behavio*” OR “sustainable consumption” OR “green consumer behavio*” OR “ecological behavio*” OR “environment* friendly consumer behavio*” OR conservationism OR “environment protect* behavio*” OR “green consumption” OR “nature protective behavio*”) AND TITLE-ABS(intervention OR experiment OR randomized OR crossover OR “pre post” OR comparison OR controlled OR control)) |

The planned search for the WEB of Science database.

| **Search string** |
| --- |
| (AB=(“natur* environment*” OR “natur* setting*” OR “natural outdoor environment*” OR “exposure to natur*” OR “nature exposure” OR “contact with natur*” OR “nature interaction*” OR “nature experience*” OR “nature assisted” OR “nature based” OR “nature therapy” OR ecotherapy OR “therapeutic landscape*” OR “time in natur*” OR “time spent in natur*” OR park OR parks OR forest OR forests OR wood OR woods OR garden OR gardens OR horticultur* OR outdoor* OR outside OR “green space*” OR greenspace* OR greenness OR “green setting*” OR “blue space*” OR seaside OR “sea side” OR wilderness OR vegetation OR tree* OR bush* OR woodland* OR allotment* OR countryside OR “nature school*” OR “experience* with natur*” OR “natural world” OR “nature contact” OR “engagement with natur*”) AND AB=(“environmental behavio*” OR “environmental action*” OR “environmentally responsible behavio*” OR “ecologically responsible behavio*” OR “environment* friendly behavio*” OR “sustainable behavio*” OR “sustainable action*” OR “pro environmental collective action*” OR “environmental activism” OR “environmental stewardship” OR “conservation behavio*” OR “environmental consumer behavio*” OR “ecological consumer behavio*” OR “sustainable consumption” OR “green consumer behavio*” OR “ecological behavio*” OR “environment* friendly consumer behavio*” OR conservationism OR “environment protect* behavio*” OR “green consumption” OR “nature protective behavio*”) AND AB=( intervention OR experiment OR randomized OR crossover OR “pre post” OR comparison OR controlled OR control)) |

The planned search for the PubMed database.

| **#** | **Search string** |
| --- | --- |
| 1 | ("Parks, Recreational"[Mesh] OR "Forests"[Mesh] OR "Gardens"[Mesh] OR "Horticulture"[Mesh] OR "Wilderness"[Mesh] OR "Trees"[Mesh]) |
| 2 | (“natur* environment*”[Title/Abstract] OR “natur* setting*”[Title/Abstract] OR “natural outdoor environment*”[Title/Abstract] OR “exposure to natur*”[Title/Abstract] OR “nature exposure”[Title/Abstract] OR “contact with natur*”[Title/Abstract] OR “nature interaction*”[Title/Abstract] OR “nature experience*”[Title/Abstract] OR “nature assisted”[Title/Abstract] OR “nature based”[Title/Abstract] OR “nature therapy”[Title/Abstract] OR ecotherapy[Title/Abstract] OR “therapeutic landscape*”[Title/Abstract] OR “time in natur*”[Title/Abstract] OR “time spent in natur*”[Title/Abstract] OR park[Title/Abstract] OR parks[Title/Abstract] OR forest[Title/Abstract] OR forests[Title/Abstract] OR wood[Title/Abstract] OR woods[Title/Abstract] OR garden[Title/Abstract] OR gardens[Title/Abstract] OR horticultur*[Title/Abstract] OR outdoor*[Title/Abstract] OR outside[Title/Abstract] OR “green space*”[Title/Abstract] OR greenspace*[Title/Abstract] OR greenness[Title/Abstract] OR “green setting*”[Title/Abstract] OR “blue space*”[Title/Abstract] OR seaside[Title/Abstract] OR “sea side”[Title/Abstract] OR wilderness[Title/Abstract] OR vegetation[Title/Abstract] OR tree*[Title/Abstract] OR bush*[Title/Abstract] OR woodland*[Title/Abstract] OR allotment*[Title/Abstract] OR countryside[Title/Abstract] OR “nature school*”[Title/Abstract] OR “experience* with natur*”[Title/Abstract] OR “natural world”[Title/Abstract] OR “nature contact”[Title/Abstract] OR “engagement with natur*”[Title/Abstract]) |
| 3 | ("Conservation of Natural Resources"[Mesh]) |
| 4 | (“environmental behavio*”[Title/Abstract] OR “environmental action*”[Title/Abstract] OR “environmentally responsible behavio*”[Title/Abstract] OR “ecologically responsible behavio*”[Title/Abstract] OR “environment* friendly behavio*”[Title/Abstract] OR “sustainable behavio*”[Title/Abstract] OR “sustainable action*”[Title/Abstract] OR “pro environmental collective action*”[Title/Abstract] OR “environmental activism”[Title/Abstract] OR “environmental stewardship”[Title/Abstract] OR “conservation behavio*”[Title/Abstract] OR “environmental consumer behavio*”[Title/Abstract] OR “ecological consumer behavio*”[Title/Abstract] OR “sustainable consumption”[Title/Abstract] OR “green consumer behavio*”[Title/Abstract] OR “ecological behavio*”[Title/Abstract] OR “environment* friendly consumer behavio*”[Title/Abstract] OR conservationism[Title/Abstract] OR “environment protect* behavio*”[Title/Abstract] OR “green consumption”[Title/Abstract] OR “nature protective behavio*”[Title/Abstract]) |
| 5 | ("Controlled Clinical Trial"[Publication Type] OR "Non-Randomized Controlled Trials as Topic"[Mesh] OR "Randomized Controlled Trials as Topic"[Mesh] OR "Control Groups"[Mesh] OR "Random Allocation"[Mesh] OR "Cross-Over Studies"[Mesh] OR "Controlled Before-After Studies"[Mesh]) |
| 6 | (intervention[Title/Abstract] OR experiment[Title/Abstract] OR randomized[Title/Abstract] OR crossover[Title/Abstract] OR “pre post”[Title/Abstract] OR comparison[Title/Abstract] OR controlled[Title/Abstract] OR control[Title/Abstract]) |
| 7 | humans[mh] |
| 8 | #1 OR #2 |
| 9 | #3 OR #4 |
| 10 | #5 OR #6 |
| 11 | #8 AND #9 AND #10 AND #7 |

The planned search for the APA PsyArticles database (via EBSCOhost).

| **Search string** |
| --- |
| (((DE "Nature (Environment)") OR AB (“natur* environment*” OR “natur* setting*” OR “natural outdoor environment*” OR “exposure to natur*” OR “nature exposure” OR “contact with natur*” OR “nature interaction*” OR “nature experience*” OR “nature assisted” OR “nature based” OR “nature therapy” OR ecotherapy OR “therapeutic landscape*” OR “time in natur*” OR “time spent in natur*” OR park OR parks OR forest OR forests OR wood OR woods OR garden OR gardens OR horticultur* OR outdoor* OR outside OR “green space*” OR greenspace* OR greenness OR “green setting*” OR “blue space*” OR seaside OR “sea side” OR wilderness OR vegetation OR tree* OR bush* OR woodland* OR allotment* OR countryside OR “nature school*” OR “experience* with natur*” OR “natural world” OR “nature contact” OR “engagement with natur*”)) AND ((DE "Pro Environmental Behavior" OR DE "Conservation (Ecological Behavior)") OR AB (“environmental behavio*” OR “environmental action*” OR “environmentally responsible behavio*” OR “ecologically responsible behavio*” OR “environment* friendly behavio*” OR “sustainable behavio*” OR “sustainable action*” OR “pro environmental collective action*” OR “environmental activism” OR “environmental stewardship” OR “conservation behavio*” OR “environmental consumer behavio*” OR “ecological consumer behavio*” OR “sustainable consumption” OR “green consumer behavio*” OR “ecological behavio*” OR “environment* friendly consumer behavio*” OR conservationism OR “environment protect* behavio*” OR “green consumption” OR “nature protective behavio*”)) AND ((DE "Intervention" OR DE "Experimental Design" OR DE "Experimental Methods" OR DE "Quasi Experimental Methods" OR DE "Randomized Controlled Trials") OR AB (intervention OR experiment OR randomized OR crossover OR “pre post” OR comparison OR controlled OR control))) |

*Note:* Search limited to the Population group of humans.

The planned search for the ERIC database (via EBSCOhost).

| **Search string** |
| --- |
| (((DE "Horticulture") OR AB (“natur* environment*” OR “natur* setting*” OR “natural outdoor environment*” OR “exposure to natur*” OR “nature exposure” OR “contact with natur*” OR “nature interaction*” OR “nature experience*” OR “nature assisted” OR “nature based” OR “nature therapy” OR ecotherapy OR “therapeutic landscape*” OR “time in natur*” OR “time spent in natur*” OR park OR parks OR forest OR forests OR wood OR woods OR garden OR gardens OR horticultur* OR outdoor* OR outside OR “green space*” OR greenspace* OR greenness OR “green setting*” OR “blue space*” OR seaside OR “sea side” OR wilderness OR vegetation OR tree* OR bush* OR woodland* OR allotment* OR countryside OR “nature school*” OR “experience* with natur*” OR “natural world” OR “nature contact” OR “engagement with natur*”)) AND ((DE "Conservation (Environment)") OR AB (“environmental behavio*” OR “environmental action*” OR “environmentally responsible behavio*” OR “ecologically responsible behavio*” OR “environment* friendly behavio*” OR “sustainable behavio*” OR “sustainable action*” OR “pro environmental collective action*” OR “environmental activism” OR “environmental stewardship” OR “conservation behavio*” OR “environmental consumer behavio*” OR “ecological consumer behavio*” OR “sustainable consumption” OR “green consumer behavio*” OR “ecological behavio*” OR “environment* friendly consumer behavio*” OR conservationism OR “environment protect* behavio*” OR “green consumption” OR “nature protective behavio*”)) AND ((DE "Intervention" OR DE "Quasiexperimental Design" OR DE "Control Groups" OR DE "Experimental Groups" OR DE "Randomized Controlled Trials") OR AB (intervention OR experiment OR randomized OR crossover OR “pre post” OR comparison OR controlled OR control))) |

The planned search for the Education Source database (via EBSCOhost).

| **Search string** |
| --- |
| (((DE "Nature study" OR DE "Parks" OR DE "Outdoor learning laboratories" OR DE "School gardens" OR DE "Outward bound schools") OR AB (“natur* environment*” OR “natur* setting*” OR “natural outdoor environment*” OR “exposure to natur*” OR “nature exposure” OR “contact with natur*” OR “nature interaction*” OR “nature experience*” OR “nature assisted” OR “nature based” OR “nature therapy” OR ecotherapy OR “therapeutic landscape*” OR “time in natur*” OR “time spent in natur*” OR park OR parks OR forest OR forests OR wood OR woods OR garden OR gardens OR horticultur* OR outdoor* OR outside OR “green space*” OR greenspace* OR greenness OR “green setting*” OR “blue space*” OR seaside OR “sea side” OR wilderness OR vegetation OR tree* OR bush* OR woodland* OR allotment* OR countryside OR “nature school*” OR “experience* with natur*” OR “natural world” OR “nature contact” OR “engagement with natur*”)) AND ((DE "Conservation of natural resources") OR AB (“environmental behavio*” OR “environmental action*” OR “environmentally responsible behavio*” OR “ecologically responsible behavio*” OR “environment* friendly behavio*” OR “sustainable behavio*” OR “sustainable action*” OR “pro environmental collective action*” OR “environmental activism” OR “environmental stewardship” OR “conservation behavio*” OR “environmental consumer behavio*” OR “ecological consumer behavio*” OR “sustainable consumption” OR “green consumer behavio*” OR “ecological behavio*” OR “environment* friendly consumer behavio*” OR conservationism OR “environment protect* behavio*” OR “green consumption” OR “nature protective behavio*”)) AND ((DE "Experimental design" OR DE "Control groups" OR DE "Experimental Groups") OR AB (intervention OR experiment OR randomized OR crossover OR “pre post” OR comparison OR controlled OR control))) |

The planned search for the GreenFILE database (via EBSCOhost).

| **Search string** |
| --- |
| (((DE "NATURE study" OR DE "NATURE parks" OR DE "NATURE centers" OR DE "FOREST reserves" OR DE "FOREST plants" OR DE "WILDERNESS areas" OR DE "TREES" OR DE "GARDENS" OR DE "HORTICULTURE" OR DE "VEGETATION greenness" OR DE "SEASHORE") OR AB (“natur* environment*” OR “natur* setting*” OR “natural outdoor environment*” OR “exposure to natur*” OR “nature exposure” OR “contact with natur*” OR “nature interaction*” OR “nature experience*” OR “nature assisted” OR “nature based” OR “nature therapy” OR ecotherapy OR “therapeutic landscape*” OR “time in natur*” OR “time spent in natur*” OR park OR parks OR forest OR forests OR wood OR woods OR garden OR gardens OR horticultur* OR outdoor* OR outside OR “green space*” OR greenspace* OR greenness OR “green setting*” OR “blue space*” OR seaside OR “sea side” OR wilderness OR vegetation OR tree* OR bush* OR woodland* OR allotment* OR countryside OR “nature school*” OR “experience* with natur*” OR “natural world” OR “nature contact” OR “engagement with natur*”)) AND ((DE "CONSERVATION of natural resources" OR DE "SUSTAINABLE consumption" OR DE "ENVIRONMENTAL activism") OR AB (“environmental behavio*” OR “environmental action*” OR “environmentally responsible behavio*” OR “ecologically responsible behavio*” OR “environment* friendly behavio*” OR “sustainable behavio*” OR “sustainable action*” OR “pro environmental collective action*” OR “environmental activism” OR “environmental stewardship” OR “conservation behavio*” OR “environmental consumer behavio*” OR “ecological consumer behavio*” OR “sustainable consumption” OR “green consumer behavio*” OR “ecological behavio*” OR “environment* friendly consumer behavio*” OR conservationism OR “environment protect* behavio*” OR “green consumption” OR “nature protective behavio*”)) AND AB (intervention OR experiment OR randomized OR crossover OR “pre post” OR comparison OR controlled OR control)) |

The planned search for the OpenDissertations database (via EBSCOhost).

| **Search string** |
| --- |
| (AB (“natur* environment*” OR “natur* setting*” OR “natural outdoor environment*” OR “exposure to natur*” OR “nature exposure” OR “contact with natur*” OR “nature interaction*” OR “nature experience*” OR “nature assisted” OR “nature based” OR “nature therapy” OR ecotherapy OR “therapeutic landscape*” OR “time in natur*” OR “time spent in natur*” OR park OR parks OR forest OR forests OR wood OR woods OR garden OR gardens OR horticultur* OR outdoor* OR outside OR “green space*” OR greenspace* OR greenness OR “green setting*” OR “blue space*” OR seaside OR “sea side” OR wilderness OR vegetation OR tree* OR bush* OR woodland* OR allotment* OR countryside OR “nature school*” OR “experience* with natur*” OR “natural world” OR “nature contact” OR “engagement with natur*”) AND AB (“environmental behavio*” OR “environmental action*” OR “environmentally responsible behavio*” OR “ecologically responsible behavio*” OR “environment* friendly behavio*” OR “sustainable behavio*” OR “sustainable action*” OR “pro environmental collective action*” OR “environmental activism” OR “environmental stewardship” OR “conservation behavio*” OR “environmental consumer behavio*” OR “ecological consumer behavio*” OR “sustainable consumption” OR “green consumer behavio*” OR “ecological behavio*” OR “environment* friendly consumer behavio*” OR conservationism OR “environment protect* behavio*” OR “green consumption” OR “nature protective behavio*”) AND AB (intervention OR experiment OR randomized OR crossover OR “pre post” OR comparison OR controlled OR control)) |
